# Association of Medical Officers of the Army and Navy of the Confederacy

**Published:** 1902-03

**Authors:** Deering J. Roberts

**Affiliations:** Secretary; Nashville, Tenn.


### Society Notes.

### Association of Medical Officers of the Army and Navy of the Confederacy.

NASHVILLE, TENN., February 25, 1902.

MY DEAR DOCTOR:—As Secretary of the Association of Medical Officers of the Army and Navy of the Confederacy, and as an ex-surgeon of the C. S. A., permit me to earnestly urge you to attend the meeting of our Association at Dallas, Texas, April 22-25, prox.

Also, allow me to request that you prepare a short paper relating to some one or more of the interesting events bearing on your professional duties in the late war between the States. If you cannot be present at the meeting, your paper would be appreciated, if sent to me by mail, although I know it would be far more agreeable and enjoyable to those who may be present, to have you with them. If you can bring or send a paper or essay, please inform me by mail within the next thirty days, giving title of paper so that it may be included in program, and greatly oblige.

Very truly and sincerely yours,

DEERING J. ROBERTS, M. D., Secretary.

The above is addressed to ex-Confederate medical officers everywhere.

